# Correlations Between OCTA Parameters and Clinical Changes in Patients Newly Diagnosed with Multiple Sclerosis

**DOI:** 10.3390/diagnostics16060828

**Published:** 2026-03-11

**Authors:** Ion Iulian Enache, Vlad Eugen Tiu, Cătălina Andreea Anghel, Alina Popa Cherecheanu, Mihai Bostan, Jacqueline Chua, Chi Li, Jia Wei Cheong, Leopold Schmetterer, Cristina Tiu

**Affiliations:** 1 Neurology Department, University Emergency Hospital Bucharest, 050098 Bucharest, Romania; ion-iulian.enache@drd.umfcd.ro (I.I.E.); cristinatiu@yahoo.com (C.T.); 2 Department of Clinical Neurosciences, Carol Davila University of Medicine and Pharmacy, 050474 Bucharest, Romania; 3 Neurology Department, Elias University Emergency Hospital, 011461 Bucharest, Romania; catalina-andreea.anghel@rez.umfcd.ro; 4 Ophthalmology Department, Carol Davila University of Medicine and Pharmacy, 050474 Bucharest, Romania; 5 Ophthalmology Department, University Emergency Hospital Bucharest, 050098 Bucharest, Romania; 6 Singapore Eye Research Institute, Singapore National Eye Centre, Singapore 169856, Singapore; 7 Ophthalmology and Visual Sciences Academic Clinical Program, Duke-NUS Medical School, National University of Singapore, Singapore 117575, Singapore; 8 SERI-NTU Advanced Ocular Engineering (STANCE), Singapore 637553, Singapore; 9 School of Chemistry, Chemical Engineering and Biotechnology, Nanyang Technological University, Singapore 637616, Singapore; 10 Department of Clinical Pharmacology, Medical University Vienna, 1090 Vienna, Austria; 11 Center for Medical Physics and Biomedical Engineering, Medical University Vienna, 1090 Vienna, Austria; 12 Aier Hospital Group, Changsha 410015, China; 13 Fondation Ophtalmologique Adolphe De Rothschild, 75019 Paris, France

**Keywords:** OCT, OCTA, biomarker, multiple sclerosis, retinal microvascular changes, SCP, DCP, choriocapillaris, RNFL, GCIPL

## Abstract

**Background:** The eye has shown potential as a reliable, readily accessible and clinically relevant site for investigating patients with multiple sclerosis (pwMS). Optical coherence tomography angiography (OCTA) shows promise in revealing new metabolic and vascular elements driving multiple sclerosis (MS) disease pathology. This study aimed to explore correlations between OCTA parameters and clinical characteristics in newly diagnosed relapsing–remitting MS (RRMS) patients. **Methods:** In this cross-sectional study, forty-one newly diagnosed RRMS patients underwent comprehensive evaluations, including neurological examinations, functional and cognitive tests (9-Hole Peg Test, Montreal Cognitive Assessment), and OCT/OCTA scans. Multiple regression analyses assessed correlations between OCT/OCTA parameters and baseline clinical characteristics. **Results:** Lower superficial capillary plexus (SCP) vessel density was associated with longer disease duration, higher EDSS scores (visual, pyramidal, cerebellar, ambulation), and impaired 9-Hole Peg Test performance, especially in the non-dominant hand. Higher values of choriocapillaris (CC) flow voids correlated with worse cognitive performance (MoCA). Structural OCT parameters showed limited clinical correlations. **Conclusions:** OCTA-derived parameters are associated with disability, fine motor function, and cognitive performance in newly diagnosed RRMS patients without prior ON. These findings suggest that retinal vascular alterations may reflect early neurodegenerative processes and provide complementary information beyond structural OCT metrics. OCTA may represent a sensitive, non-invasive imaging biomarker for patient assessment in early MS.

## 1. Introduction

The eye has shown potential as a reliable, readily accessible and clinically relevant site for investigating patients with multiple sclerosis (pwMS), as supported by both functional and structural data [1,2,3,4]. Reflecting this, the 2024 revision of the McDonald diagnostic criteria now includes the optic nerve as the fifth topographic site for multiple sclerosis (MS) lesions [5].

Involvement of the optic nerve is frequent in MS, with optic neuritis (ON) reported in around 70% of patients [6]. Additional independent and often subclinical chronic visual disturbances widen the spectrum of ophthalmologic manifestations in MS. These are most probably secondary to neurodegeneration and progressive axonal loss [7], which may actually be a key driver in the long term. Optical coherence tomography (OCT) studies show similar rates of retinal thinning (especially at the level of the retinal nerve fiber layer (RNFL)), regardless of previous inflammatory activity, clinically manifested as acute ON [8].

Recent data indicates that the main driver of disability accrual in pwMS is progression independent of relapse activity (PIRA), taking place from the earliest disease stages [9]. Whether PIRA and chronic retinal degeneration share the same mechanisms remains unclear.

**Figure 1:**
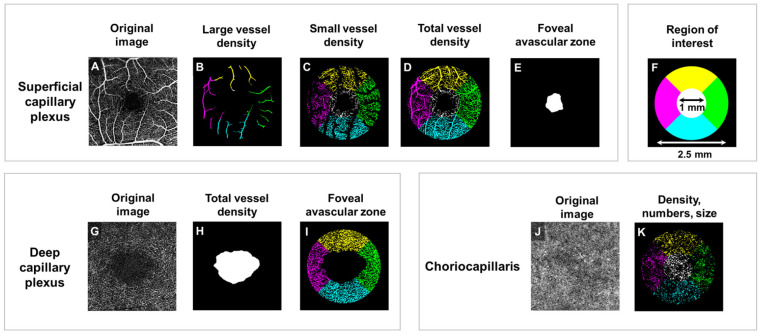
Optical coherence tomography angiography (OCTA) analysis of retinal vasculature. (**A**–**F**) Superficial capillary plexus (SCP): (**A**) Original OCTA image of the SCP showing detailed retinal vasculature. (**B**) Large vessel density map, highlighting major retinal vessels. (**C**) Small vessel density map, showcasing finer capillary networks. (**D**) Total vessel density map, combining large and small vessel information. (**E**) Foveal avascular zone (FAZ), illustrating the central area devoid of capillaries. (**F**) Schematic representation of the region of interest, demonstrating the 1 mm and 2.5 mm diameter circles used for quantitative analysis. (**G**–**I**) Deep capillary plexus (DCP): (**G**) Original OCTA image of the DCP, revealing deeper retinal vascular structures. (**H**) Total vessel density map for the DCP, quantifying overall vascular density. (**I**) FAZ in the DCP, showing the central avascular area. (**J**,**K**) Choriocapillaris: (**J**) Original OCTA image of the choriocapillaris, depicting the vascular layer supplying the outer retina. (**K**) Density, numbers, and size analysis of choriocapillaris, illustrating the quantification of vascular characteristics.

Using a similar technique to ultrasound imaging [10], optical coherence tomography angiography (OCTA) allows the visualization of the main plexuses involved in retinal vascular supply: the superficial capillary plexus (SCP)—serving the inner retina, including the RNFL and ganglion cell layer–inner plexiform layer (GCIPL); the deep capillary plexus (DCP)—serving the inner nuclear layer (INL) and outer plexiform layer (OPL); and the choriocapillaris—contributing to the vascularization of the outermost retinal layers, as well as microcirculation surrounding the optic nerve head [11] (Figure 1).

As the retina is one of the most metabolically active structures of the human body, its vascular supply has developed the capacity to rapidly adapt to the constant fluctuations in energy demand [12]. The ability to detect such changes could provide valuable insights into early alterations occurring in pwMS, with important implications regarding disease diagnosis, prognosis and management.

Blood–brain barrier disruption is an early pathological feature in MS. Even though it has been classically viewed as a secondary effect of the focal neuroinflammatory lesions, emerging evidence supports the role of primary vascular changes as a standalone mechanism [13].

Our study aims to explore the correlations between OCTA parameters and clinical characteristics of pwMS to better assess the added value of vascular retinal imaging at baseline compared with conventional OCT imaging metrics.

## 2. Materials and Methods

We designed a cross-sectional study that included patients newly diagnosed with relapsing–remitting multiple sclerosis (RRMS) in the previous 6 months (according to the 2017 McDonald criteria) [14] in the Multiple Sclerosis Center of the University Emergency Hospital, Bucharest, Romania, from June 2020 to October 2021. Inclusion criteria required a minimum age of 18 years. The main exclusion criteria were ongoing pregnancy or medical history that could interfere with the study protocol, such as any clinically relevant ocular pathology (detailed in Supplementary Material File S1). Eyes with a history of ON were excluded from the study.

The study protocol was approved by the Ethics Committee of the University Emergency Hospital Bucharest (Registration number 12525/6), and all patients were required to sign a written informed consent prior to inclusion. After quality analysis and application of exclusion criteria, the final study cohort consisted of 41 patients. The sample size reflects all eligible patients recruited during the study period.

### 2.1. Study Protocol

The study protocol, detailed in Supplementary Material File S1, included a baseline evaluation with clinical testing, serum and cerebrospinal fluid (CSF) sampling, contrast enhanced cerebral MRI, and OCT and OCTA scans. Clinical assessments included neurological examination, standardized functional and cognitive tests, as well as disability predictive scores. All patients were treatment-naïve at the time of inclusion and had not initiated immunomodulatory or other disease-modifying therapies (DMTs) prior to baseline evaluation.

### 2.2. OCT and OCTA Acquisition Protocol

All OCT and OCTA scans were performed within the Ophthalmology Department of the University Emergency Hospital of Bucharest. Each participant received two 6 × 6 mm OCT scans, one centered on the optic disc and the other centered on the macula, and a macula-centered 3 × 3 mm OCTA scan. Each OCTA scan was then automatically segmented into vascular plexuses (SCP, DCP and choriocapillaris) and subsequently loaded into a customized MATLAB algorithm (MATLAB R2024a, MathWorks, Natick, MA, USA) to evaluate the capillary densities (Figure 1). The detailed protocol regarding OCTA segmentation can be found in Supplementary File S1 and Figure S1. For patients with unilateral ON, the allocated OCT and OCTA measurements only included those from the unaffected eye, while for the patients without history of ON the allocated OCT and OCTA measurements were represented by the mean value between the two unaffected eyes. In order to eliminate possible changes resulting from neuroinflammatory activity, we excluded from the analysis the eyes that presented clinical events suggestive of ON, or changes in the OCT parameters suggestive of a history of asymptomatic ON (inter-eye difference in RNFL thickness > 5 µm, or in GCIPL thickness > 4 µm) [15].

### 2.3. Statistical Analysis

Statistical analysis was conducted using R version 4.0.4 (The R Foundation for Statistical Computing, Vienna, Austria). Multivariable linear regression models were employed, adjusting for age and gender as covariates. For each model, the β coefficient (effect size), 95% confidence interval (CI), and *p*-value were estimated, with statistical significance set at *p* < 0.05.

## 3. Results

Table 1 shows the main demographic and clinical characteristics of the study cohort. Table 2 and Table 3 summarize the results described and discussed below. The results in their extensive format can be consulted in Supplementary Material File S2. All the assessments, including OCT/OCTA acquisitions, were performed before the initiation of any DMT.

### 3.1. Disease Duration

No significant correlations were found between OCT structural metrics (RNFL: *p* = 0.256, GCIPL: *p* = 0.420) and disease duration. From the OCTA parameters, SCP VD showed negative correlation with disease duration, with small vessel SCP VD (β = −0.674, 95% CI: −1.208 to −0.140, *p* = 0.018) and total SCP VD (β = −0.725, 95% CI: −1.204 to −0.247, *p* = 0.005) being lower in individuals with longer disease duration. Deep FAZ area was positively correlated with disease duration (β = 2.448, 95% CI: 0.131 to 4.765, *p* = 0.045).

### 3.2. Disability Status (EDSS)

Among OCT structural metrics, GCIPL thickness was negatively correlated with the Expanded Disability Status Scale (EDSS) (β = −0.047, 95% CI: −0.091 to −0.003, *p* = 0.041). Among OCTA vascular metrics, only large vessel SCP VD demonstrated a significant negative correlation with EDSS (β = −1.545, 95% CI: −2.582 to −0.508, *p* = 0.006).

When specifically analyzing visual EDSS scores, among OCT structural metrics, neither RNFL (*p* = 0.762) nor GCIPL (*p* = 0.107) showed significant correlations. Among OCTA vascular metrics, both small vessel SCP VD (β = −0.307, 95% CI: −0.467 to −0.148, *p* = 0.001) and total SCP VD (β = −0.265, 95% CI: −0.415 to −0.114, *p* = 0.001) were significantly negatively correlated with visual EDSS scores. Additionally, total DCP VD showed a significant negative correlation with the visual EDSS score (β = −0.127, 95% CI: −0.249 to −0.005, *p* = 0.047). Among choriocapillaris flow void metrics, flow void size was positively correlated with the visual EDSS score (β = 0.006, 95% CI: 0.001 to 0.011, *p* = 0.032). For FAZ metrics, both superficial FAZ perimeter (β = 0.929, 95% CI: 0.233 to 1.625, *p* = 0.013) and superficial FAZ circularity (β = 4.391, 95% CI: 1.194 to 7.588, *p* = 0.011) showed significant positive correlations with visual EDSS scores. Additionally, deep FAZ area (β = 0.859, 95% CI: 0.113 to 1.605, *p* = 0.030) and deep FAZ perimeter (β = 0.480, 95% CI: 0.107 to 0.852, *p* = 0.016) were also significantly correlated.

In order to assess the correlation between OCT/OCTA parameters and EDSS scores independently of the effect of the visual EDSS score, we conducted a subgroup analysis of the patients with a visual EDSS of 0 points. The correlation between large vessel density at the level of the SCP and EDSS scores observed in the full cohort persisted in this subgroup of patients (β = −1.574, 95% CI: −2.864 to −0.284, *p* = 0.024), suggesting that lower large VD at the level of the SCP correlates with higher EDSS scores independently of visual function. Among OCT structural metrics, both RNFL (β = −0.056, 95% CI: −0.099 to −0.013, *p* = 0.019) and GCIPL (β = −0.099, 95% CI: −0.160 to −0.038, *p* = 0.004) showed significant correlation with EDSS scores in this subgroup.

We further analyzed the correlation between each parameter of the EDSS score and SCP VD. Higher pyramidal EDSS scores correlated with lower large vessel SCP VD (β = −0.935, 95% CI: −1.752 to −0.118, *p* = 0.031). Cerebellar EDSS scores negatively correlated with total SCP VD (β = −0.239, 95% CI: −0.410 to −0.068, *p* = 0.009), small vessel VD (β = −0.239, 95% CI: −0.410 to −0.068, *p* = 0.009) and large vessel VD (β = −0.944, 95% CI: −1.775 to −0.113, *p* = 0.032). Ambulation scores also presented significant negative correlations involving all vessels at the level of the SCP (total vessels: β = −0.383, 95% CI: −0.659 to −0.107, *p* = 0.010, small vessels: β = −0.324, 95% CI: −0.636 to −0.012, *p* = 0.049, and large vessels: β = −1.920, 95% CI: −3.216 to −0.624, *p* = 0.006). No other significant correlations were found between SCP VD and other subunits of the EDSS score.

### 3.3. Clinical Functional Testing

Among OCT structural metrics, neither RNFL (*p* = 0.095) nor GCIPL (*p* = 0.657) were significantly correlated with 9HPT performance in the non-dominant hand. Small vessel SCP VD (β = −1.568, 95% CI: −2.414 to −0.722, *p* = 0.001) and total SCP VD (β = −1.491, 95% CI: −2.264 to −0.718, *p* = 0.001) were significantly lower in individuals with slower 9HPT performance. For choriocapillaris flow voids, both flow void number (β = −0.018, 95% CI: −0.032 to −0.004, *p* = 0.013) and flow void size (β = 0.045, 95% CI: 0.019 to 0.071, *p* = 0.002) were significantly correlated with 9HPT time. Superficial FAZ circularity (β = 18.349, 95% CI: 0.947 to 35.751, *p* = 0.046) was significantly correlated with 9HPT performance. In the dominant hand, similar trends were observed, though correlations were generally weaker. Total SCP VD (β = −0.666, 95% CI: −1.292 to −0.040, *p* = 0.044) showed a significant correlation. Choriocapillaris flow void number (*p* = 0.033) and size (*p* = 0.003) were also significantly correlated. There was no significant correlation involving 25-Foot Walk Test (25FWT) times.

### 3.4. Cognitive Assessment

Larger choriocapillaris flow void size (β = −0.030, 95% CI: −0.056 to −0.004, *p* = 0.032) was the only parameter significantly correlated with lower Montreal Cognitive Assessment (MoCA) scores at baseline. Choriocapillaris flow void number (β = 0.046, 95% CI: 0.004 to 0.088, *p* = 0.037) was the only parameter significantly correlated with Single Digit Modalities Test (SDMT) scores.

### 3.5. Predictive Scores

GCIPL (β = −0.040, 95% CI: −0.075 to −0.005, *p* = 0.029) was significantly correlated with risk of ambulatory disability (RoAD) scores [16] (Annex S1 in Supplementary File S3). No significant correlations were observed for other OCT and OCTA metrics. There were no significant correlations involving Bayesian Risk Estimate for MS at Onset (BREMSO) scores [17] (Annex S2 in Supplementary File S3) at baseline.

## 4. Discussion

The Optical Coherence Tomography in Multiple Sclerosis (OCTiMS) study, the first large prospective multicenter study assessing OCT alterations in pwMS, reported significant thinning of the inner retinal layers compared to controls, irrespective of a history of MS-associated clinical ON [8]. These results indicate that OCT measurements may detect not only neuroinflammatory processes, but also neuroaxonal degeneration. As chronic inflammation and progressive degeneration have been demonstrated to be the underlying cause of PIRA, the main driver of disability accrual in pwMS [9], OCT could prove to be a valuable biomarker of disease progression.

The OCTiMS study reported faster RNFL and GCIPL thinning in patients with shorter disease durations (<3 years), compared to those with longer disease durations (>5 years) [8]. These changes were attributed to either a greater dynamic range of the retinal layers in the first stages of the disease, or a faster RNFL loss due to more active inflammation in early stages. In our cohort, there were no significant correlations between OCT-derived metrics and disease duration. However, although the median disease duration was 1 year, we observed a broad range of RNFL and GCIPL values, reflecting the heterogeneity in retinal neuroaxonal integrity already present at diagnosis. Nevertheless, the detection of OCT alterations at this stage may be limited due to only subtle structural changes, which may progress rapidly afterwards.

A significant correlation was observed between disease duration and VD deficit at the level of the SCP. These findings suggest that microvascular changes could have a faster dynamic than those detected by classical OCT imaging, potentially preceding or even contributing to subsequent neuroaxonal loss. It is still a matter of debate whether the retinal vascular alterations represent secondary adaptation to reduced metabolic needs of the retinal layers caused by atrophy, or rather a primary cause of energy failure through hypoperfusion and hypoxia [11,12,18]. One study suggests that those two processes could be intertwined, each contributing to changes in different sites. When comparing MS patients with ON (MS-ON) to MS-NON patients, the authors noted a reduction in medium-sized vessels of the SCP, which was strongly linked to GCIPL thinning, thus implying that the vascular changes occurred secondarily to the reduced metabolic demands of the GCIPL. Both MS-ON and MS-NON patients presented small vessel alterations of the SCP, independent of GCIPL thickness, which could indicate that retinal microcirculation alterations could be caused by disruptions of the blood–retinal barrier [19].

Previous studies showed that the EDSS score correlates with structural retinal parameters detected by OCT [20,21]. Data regarding OCTA measurements are very scarce. A study published by Lang et al. in 2023 on OCTA findings in NMOSD and MOGAD patients reported significant correlations between the SCP and the EDSS scores in those patients [22]. However, these findings were not validated for pwMS. In our cohort, both GCIPL thickness and large vessel SCP VD demonstrated a significant negative correlation with EDSS scores. The correlations between OCT parameters, SCP VD (OCTA parameter) and EDSS scores persisted in the subgroup of patients with a visual EDSS of 0 points.

Many additional correlations involving OCTA parameters were found when specifically analyzing visual EDSS. These changes suggest a global involvement of the SCP and DCP, with higher visual scores correlated with lower vessel densities. While the involvement of the SCP has been consistently reported in OCTA studies in pwMS despite variability in study protocols and findings [23], data regarding DCP alterations is scarce and inconclusive [24]. In contrast, RNFL and GCIPL thicknesses showed no significant correlation with visual EDSS scores in our cohort. While there is a paucity of published data regarding correlations between OCT parameters and clinical visual status in eyes without a history of ON, small studies seem to confirm our findings that lower RNFL does not directly correlate with lower visual performance when excluding ON [20].

Pyramidal, cerebellar and ambulation scores proved to have multiple significant correlations involving both large and small vessel densities at the level of the SCP. Previous studies demonstrated that both cerebellar and corticospinal tract integrity is affected by the progressive degeneration taking place since early disease stages [25,26,27]. Our findings suggest that retinal vascular changes, as expressed by VD alterations at the level of the SCP, may be a timely indicator of these global neurodegenerative processes even in recently diagnosed patients.

Times for 9HPT in the non-dominant hand showed significant negative correlations with total and small vessel SCP VD. A similar trend was observed for the dominant hand, although the correlations were generally weaker. The reason for this finding could be that fine motor control of the non-dominant hand relies more on visual cues compared to the dominant hand. Additionally, data derived from functional brain imaging shows that tasks requiring localization and visuospatial attention lateralize to the right hemisphere [28]. That could explain why a reduced vascular supply through the SCP to the directional-sensitive neurons located in the IPL could lead to decreased performance of a task requiring a high degree of visuospatial attention such as the 9HPT, especially in the left, non-dominant hand [29]. Another possibility is that performance times for the non-dominant hand may be more sensitive to subtle impairments, making it a more reliable marker of disability.

In contrast to previous findings involving the SCP and DCP, cognitive function was exclusively linked to choriocapillaris flow voids. As opposed to both SCP and DCP, the choriocapillaris is not isolated from systemic circulation by the blood–retinal barrier and it consists of a single layer of wide-diameter capillaries with a fenestrated endothelium [30]. All these factors contribute to a higher vulnerability to systemic vascular dysfunction. While cognitive impairment in MS is considered to be multifactorial, recent studies have noted a high prevalence of vascular comorbidities possibly associated with cognitive impairment in pwMS [31]. Our findings also support the contribution of a systemic vascular component to cognitive function in pwMS, offering new insights into the characteristics of cognitive impairment in this population.

Higher RoAD scores, indicative of an increased risk of severe disability at a 10-year follow-up [16], were correlated with thinner GCIPL, without any other significant correlation involving other OCT or OCTA parameters. The BREMSO score, another predictive score which is used to calculate individualized risk at baseline using clinical parameters [17], did not show any significant correlation with OCTA parameters. Consistent with the absence of correlation between OCT and OCTA parameters and 25FWT scores, our findings suggest that the early vascular changes highlighted by OCTA metrics may be linked to visual and cognitive impairment, or disability affecting fine motor tasks more heavily reliant on visual cues, rather than general disability and ambulation.

Limitations of this study are related to the small sample size and a monocentric study design. Although MRI examinations were available, this data was not analyzed due to variability in acquisition protocols and image quality, thus preventing reliable correlations with OCTA parameters. Larger samples with long-term follow-up are needed to validate these initial exploratory findings. Additionally, it is necessary to implement a standardized analysis protocol before OCTA can be fully integrated into clinical practice [32].

## 5. Conclusions

The findings of this study support the use of OCTA as a sensitive, non-invasive imaging biomarker for pwMS. One of the core strengths of this study is the large number of data collected, which allowed for the analysis of less frequently used OCTA parameters in neurological disorders, such as FAZ circularity, area and perimeter and choriocapillaris flow voids.

While the majority of the clinical data available at baseline showed no correlation with structural OCT findings, changes involving numerous OCTA metrics suggest that retinal vascular changes can potentially become a valuable biomarker of disease severity in pwMS. The study protocol only allowed for the inclusion of RRMS patients without a history of ON, aiming to focus on more subtle, progressive, and neurodegenerative changes. The correlations identified between specific OCTA metrics and various clinical characteristics could provide important insights regarding possible disease mechanisms.

## Figures and Tables

**Table 1:** Demographic data and main clinical characteristics of the study cohort.

Demographic Data	Mean/Median (Minimum and Maximum Values)
Age (years)	30 (18; 52)
Sex (female (F)/male (M))	26 F (63.41%)/15 M (36.58%)
Smoker status (active/non-smoker)	11 (26.82%)/30 (73.17%)
Lifestyle (active/sedentary)	29 (70.73%)/12 (29.26%)
Urban/rural	32 (78.04%)/9 (21.95%)
Clinical data	Median (IQR)
Disease duration (years)	1 (0–2)
EDSS	2 (1.5–2.5)
Visual EDSS	0 (0–1)
9HPT time—dominant hand (seconds)	20.63 (18.15–22.9)
9HPT time—non-dominant hand (seconds)	21.46 (19.68–23.93)
25-Foot Walk Test (seconds)	6.19 (5.69–7.02)
MoCA score (points)	26 (24–29)
SDMT score (points)	43 (36–54)
OCT characteristics	Median (IQR)
RNFL	97 µm (86–102)
GCIPL	80 µm (75–86)
Predictive scores	Mean/median (minimum and maximum values)
RoAD (points)	3 (0; 5)
BREMSO (points)	0.6 (−0.65; 2.39)

9HPT: Nine-Hole Peg Test; BREMSO: Bayesian Risk Estimate for MS at Onset; EDSS: expanded disability status score; GCIPL: ganglion cell–inner plexiform layer; IQR: interquartile range; MoCA: Montreal Cognitive Assessment; RoAD: risk of ambulatory disability score; RNFL: retinal nerve fiber layer; SDMT: Symbol Digits Modalities Test.

**Table 2:** Summary of statistically significant correlations between OCT/OCTA parameters and clinical characteristics at baseline in our study cohort.

Parameter	β	95% CI	*p* Value
Disease duration			
SCP small vessels, VD	−0.674	−1.208 to −0.140	**0.018**
SCP total vessels, VD	−0.725	−1.204 to −0.247	**0.005**
Deep FAZ area	2.448	0.131 to 4.765	**0.045**
9HPT non-dominant hand			
SCP small vessels, VD	−1.568	−2.414 to −0.722	**0.001**
SCP total vessels, VD	−1.491	−2.264 to −0.718	**0.001**
Choriocapillaris flow voids, number	−0.018	−0.032 to −0.004	**0.013**
Choriocapillaris flow voids, size	0.045	0.019 to 0.071	**0.002**
Superficial FAZ circularity	18.349	0.947 to 35.751	**0.046**
9HPT dominant hand			
SCP total vessels, VD	−0.666	−1.292 to −0.040	**0.044**
Choriocapillaris flow voids, number	−0.011	−0.021 to −0.001	**0.033**
Choriocapillaris flow voids, size	0.031	0.012 to 0.050	**0.003**
MoCA scores at baseline			
Choriocapillaris flow voids, size	−0.030	−0.056 to −0.004	**0.032**
SDMT scores at baseline			
Choriocapillaris flow voids, number	0.046	0.004 to 0.088	**0.037**
RoAD scores at baseline			
GCIPL	−0.040	−0.075 to −0.005	**0.029**

9HPT: Nine-Hole Peg Test; CI: confidence interval; GCIPL: ganglion cell–inner plexiform layer; MoCA: Montreal Cognitive Assessment; RoAD: risk of ambulatory disability score; SDMT: Symbol Digits Modalities Test; SCP = superficial capillary plexus; VD = vessel density; FAZ = foveal avascular zone. Statistically significant *p*-values (*p* < 0.05) are shown in bold.

**Table 3:** Correlations between OCT/OCTA metrics and EDSS score components.

Parameter	β	95% CI	*p* Value
EDSS Score at Baseline (Full Cohort)			
GCIPL	−0.047	−0.091 to −0.003	**0.041**
SCP large vessels, VD	−1.545	−2.582 to −0.508	**0.006**
EDSS score at baseline (subgroup of patients with 0 points on visual EDSS)			
RNFL	−0.056	−0.099 to −0.013	**0.019**
GCIPL	−0.099	−0.160 to −0.038	**0.004**
SCP large vessels, VD	−1.574	−2.864 to −0.284	**0.024**
Visual EDSS score at baseline			
SCP small vessels, VD	−0.307	−0.467 to −0.148	**0.001**
SCP total vessels, VD	−0.265	−0.415 to −0.114	**0.001**
DCP total vessels, VD	−0.127	−0.249 to −0.005	**0.047**
Choriocapillaris flow voids, size	0.006	0.001 to 0.011	**0.032**
Superficial FAZ perimeter	0.929	0.233 to 1.625	**0.013**
Superficial FAZ circularity	4.391	1.194 to 7.588	**0.011**
Deep FAZ area	0.859	0.113 to 1.605	**0.030**
Deep FAZ perimeter	0.480	0.107 to 0.852	**0.016**
Pyramidal EDSS score at baseline (SCP only)			
SCP large vessels, VD	−0.935	−1.752 to −0.118	**0.031**
Cerebellar EDSS score at baseline (SCP only)			
SCP large vessels, VD	−0.944	−1.775 to −0.113	**0.032**
SCP small vessels, VD	−0.210	−0.400 to −0.020	**0.038**
SCP total vessels, VD	−0.239	−0.410 to −0.068	**0.009**
Ambulation EDSS score at baseline (SCP only)			
SCP large vessels, VD	−1.920	−3.216 to −0.624	**0.006**
SCP small vessels, VD	−0.324	−0.636 to −0.012	**0.049**
SCP total vessels, VD	−0.383	−0.659 to −0.107	**0.010**

CI: confidence interval; EDSS: expanded disability status score; GCIPL: ganglion cell–inner plexiform layer; RNFL: retinal nerve fiber layer; SCP = superficial capillary plexus; VD = vessel density; DCP = deep capillary plexus; FAZ = foveal avascular zone. Statistically significant *p*-values (*p* < 0.05) are shown in bold.

## Data Availability

The data analyzed in this study are not publicly available due to patient confidentiality but may be provided by the corresponding author upon reasonable request.

## Supplementary Materials

The following supporting information can be downloaded at: https://www.mdpi.com/article/10.3390/diagnostics16060828/s1, File S1: Figure S1: Framework of optical coherence tomography angiography (OCTA) image post-processing; File S2: Table S1: Correlations of OCT/OCTA parameters with disease duration; Table S2: Correlations of OCT/OCTA parameters with overall EDSS scores at baseline; Table S3: Subgroup analysis of correlations of OCT/OCTA parameters with overall EDSS scores at baseline for the patients with a visual EDSS of 0 points; Table S4: Correlations of OCT/OCTA parameters with the visual component of the EDSS scores at baseline; Table S5: Correlations between vessel density at the level of the SCP and individual functional components of the EDSS score (full cohort); Table S6: Correlations of OCT/OCTA parameters with 9 hole peg test (9HPT) at baseline; Table S7: Correlations of OCT/OCTA parameters with MoCA scores at baseline; Table S8; Correlations of OCT/OCTA parameters with SDMT scores at baseline; Table S9: Correlations of OCT/OCTA parameters with RoAD scores at baseline; File S3: Annex S1: RoAD scoring at baseline; Annex S2: BREMSO scoring. Reference [33] are cited in the Supplementary Materials.

## Abbreviations

The following abbreviations are used in this manuscript:

25FWT	25-Foot Walk Test
9HPT	Nine-Hole Peg Test
BREMSO	Bayesian Risk Estimate for Multiple Sclerosis at Onset
CC	Choriocapillaris
CSF	Cerebrospinal Fluid
DCP	Deep Capillary Plexus
EDSS	Expanded Disability Status Scale
FAZ	Foveal Avascular Zone
GCIPL	Ganglion Cell Layer–Inner Plexiform Layer
INL	Inner Nuclear Layer
IPL	Inner Plexiform Layer
MRI	Magnetic Resonance Imaging
MoCA	Montreal Cognitive Assessment
MOGAD	Myelin Oligodendrocyte Glycoprotein Antibody-Associated Disease
MS	Multiple Sclerosis
MS-NON	Multiple Sclerosis without Optic Neuritis
MS-ON	Multiple Sclerosis with Optic Neuritis
NMOSD	Neuromyelitis Optica Spectrum Disorder
OCT	Optical Coherence Tomography
OCTA	Optical Coherence Tomography Angiography
ON	Optic Neuritis
OPL	Outer Plexiform Layer
PIRA	Progression Independent of Relapse Activity
pwMS	Patients with Multiple Sclerosis
RNFL	Retinal Nerve Fiber Layer
RoAD	Risk of Ambulatory Disability Score
RRMS	Relapsing–Remitting Multiple Sclerosis
SCP	Superficial Capillary Plexus
SDMT	Symbol Digit Modalities Test
VD	Vessel Density
