# Supplementary material for: Correlations Between OCTA Parameters and Clinical Changes in Patients Newly Diagnosed with Multiple Sclerosis
Source: Diagnostics (Basel). 2026 Mar 11;16(6):828. doi: 10.3390/diagnostics16060828 (PMC13025533; doi:10.3390/diagnostics16060828)
Supplement: Supplementary file 1 [file diagnostics-16-00828-s001.zip › Supplementary Material File S1.pdf]

Supplementary Material File S1 - Study Protocol

Inclusion criteria:

- minimum of 18 years of age;
- diagnosis of Relapsing–Remitting Multiple Sclerosis (RRMS) in the previous 6 months according to the 2017 McDonald criteria.

Exclusion criteria:

- ongoing pregnancy;
- any medical history that could interfere with the study protocol, such as threatening ocular diseases or any other clinically relevant ocular disease;
- for patients presenting with a history of monocular acute optic neuritis, the affected eye was excluded from the analysis;
- in patients without a history suggestive of symptomatic optic neuritis, we excluded the scans where inter-eye difference in RNFL thickness was >5 μm, or where inter-eye difference in GCIPL thickness was >4 μm, suggesting the possibility of asymptomatic optic neuritis.

Baseline evaluation:

- serum and cerebrospinal fluid (CSF) collection;
- contrast-enhanced cerebral magnetic resonance imaging (MRI);
- optical coherence tomography (OCT) and OCT angiography (see protocol below).
- Baseline clinical evaluation:
    - neurological exam;
    - Expanded Disability Status Scale (EDSS) scoring;
    - 9-hole peg test (9HPT), 25-Foot Walk Test (25FWT);
    - cognitive assessment using the Montreal Cognitive Assessment (MoCA) test and Symbol Digit Modalities Test (SDMT);
    - Predictive scores: the Risk of Ambulatory Disability (RoAD) (Annex 1) and Bayesian Risk Estimate for MS at Onset (BREMSO) (Annex 2).

OCT and OCTA scanning and processing protocol:

- all OCT and OCTA scans were performed within the Ophthalmology Department of the University Emergency Hospital of Bucharest, using a Cirrus™ AngioPlex HD-OCT 5000 Spectral-Domain OCT (Carl Zeiss Meditec, Inc.) machine;
- patients underwent two OCT scans, one centered on the optic disc (200 A-scans x 200 B-scans; 6 x 6 mm), and the other centered on the macula (512 A-scans x 128 B-scans; 6 x 6 mm);
- patients underwent one 3x3 mm^2 macula-centered OCTA scan that consisted of an isotropic sampling followed by four consecutive B-scans;
- each OCTA scan was automatically segmented into the SCP and DCP by the Cirrus Review Software, and subsequently loaded into a customized MATLAB algorithm to evaluate capillary densities;
- quality criteria included: signal strength score of 7 or above; absence of movement artifacts causing breakage or off-centration; and no algorithm failures resulting in segmentation errors;

- after quality control and application of the aforementioned exclusion criteria, each included patient was then allocated a singular value for each OCT and OCTA parameter, corresponding either to: 1. the unaffected eye if history of monocular ON is present; or 2. the mean value of the two eyes if no history of ON is present.

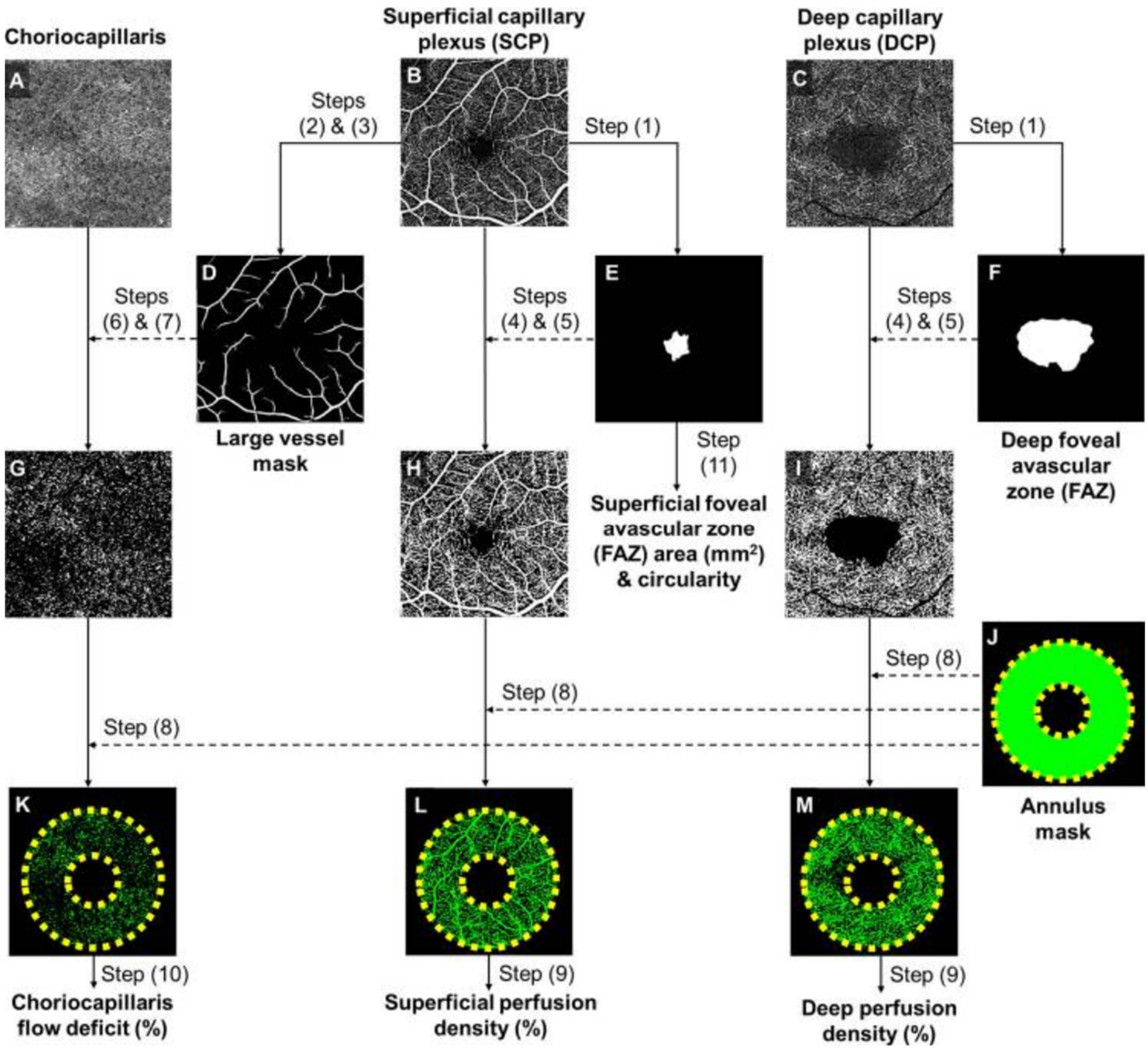

Figure S1. Framework of optical coherence tomography angiography (OCTA) image post-processing. **A–C** Raw OCTA images extracted from the OCTA machine. **D** Large vessels segmented and binarized from the superficial capillary plexus (SCP). **E–F** Foveal avascular zones (FAZs) are manually delineated from SCP and deep capillary plexus (DCP). **G** Choriocapillaris flow deficits binarized from the OCTA image by applying a mask to remove large vessel artifacts. **H-I** Vessels binarized from the SCP and DCP. FAZ regions were masked from the binarized images. **J** Fovea-centered annulus mask has an inner diameter of 1.0 mm and an outer diameter of 2.5 mm. **K** Choriocapillaris flow deficit density is obtained within the annulus mask. **L-M** Perfusion density is obtained by binarizing vascular images with an annulus mask. Dotted lines represent the overlay of masks.

Extraction of OCTA measurements [1]

Each OCTA scan was automatically segmented into the SCP, DCP, and choriocapillaris using the Cirrus Review Software (Carl Zeiss Meditec, version 11.0.0.29946). The SCP spans the inner limiting membrane (ILM) to the IPL, while the DCP spans the INL to the OPL. The choriocapillaris spans from 31 µm below the RPE to 40 µm below the RPE. Images were checked by a technician using the review software to ensure correct segmentation, and no manual adjustment was needed. Projection artifacts from the overlying retinal circulation were removed from the DCP using the software integrated with the instrument.

OCTA images of the three slabs were subsequently loaded into a customized MATLAB (The MathWorks Inc., Natick, MA) algorithm to extract the OCTA measurements (Fig. 2). The OCTA processing framework comprised the following steps: (1) outlining the foveal avascular zone (FAZ) border manually on the SCP and DCP slabs, (2) enhancing the contrast of the large vessels on the SCP with a combination of Hessian and Gabor filters, (3) then subsequently binarizing it to produce a large vessel mask; (4) binarization by setting a threshold at the mean intensity on the SCP and DCP slabs, (5) after which masking of FAZ regions; (6) large vessel artifacts were removed from the choriocapillaris slab, and (7) flow deficits were then binarized by setting a threshold that was one standard deviation below the mean intensity of the respective OCTA images; analysis (8) with a fovea-centered annulus (inner diameter 1.0 mm, outer diameter 2.5 mm) was performed to obtain the results (Fig. S1).

The evaluation of perfusion density was computed as the percentage of perfused area per total imaged area. Area and circularity of FAZ in the SCP were computed, where the circularity of the FAZ refers to the ratio between the perimeter of the FAZ and the perimeter of an equivalent circle (i.e., circle of the same area). The circularity value ranges from 0.0 to 1.0. A value closer to 0.0 indicates an irregular shape of the FAZ, while a value closer to 1.0 indicates a more circular shape. For the choriocapillaris slab, the evaluation was based on the absence of flow signals. Flow deficit density was computed as the percentage of flow deficit area per total imaged area.

Bibliography

1. Bostan M, Li C, Cheong JW, Wong DWK, Tan B, Ismail MB, Garhöfer G, Coroleuca R, Schmetterer L, Popa-Cherecheanu A, Chua J. Longitudinal analysis of retinal and choriocapillaris in patients with multiple sclerosis: a 3-year study. J Neurol. 2025 Mar 1;272(3):237. doi: 10.1007/s00415-025-12930-7. PMID: 40025301; PMCID: PMC11872746.
