# Supplementary material for: Correlations Between OCTA Parameters and Clinical Changes in Patients Newly Diagnosed with Multiple Sclerosis
Source: Diagnostics (Basel). 2026 Mar 11;16(6):828. doi: 10.3390/diagnostics16060828 (PMC13025533; doi:10.3390/diagnostics16060828)
Supplement: Supplementary file 1 [file diagnostics-16-00828-s001.zip › Supplementary Material File S2.pdf]

**Supplementary File S2** – Detailed statistical analysis results

**Table S1.** Correlations of OCT/OCTA parameters with disease duration.

| **Parameter** | **β** | **95% CI** | **p value** |
|---|---|---|---|
| **OCT** | | | |
| RNFL | 0.043 | -0.030 to 0.117 | 0.256 |
| GCIPL | -0.042 | -0.143 to 0.059 | 0.420 |
| **OCTA** | | | |
| **SCP, VD** | | | |
| Large vessels, VD | -2.449 | -4.845 to -0.052 | 0.053 |
| Small vessels, VD | -0.674 | -1.208 to -0.140 | **0.018** |
| Total vessels, VD | -0.725 | -1.204 to -0.247 | **0.005** |
| **DCP** | | | |
| Total vessels, VD | -0.253 | -0.639 to 0.133 | 0.206 |
| **Choriocapillaris flow voids** | | | |
| Choriocapillaris flow voids, density | -0.200 | -0.772 to 0.373 | 0.499 |
| Choriocapillaris flow voids, number | -0.005 | -0.013 to 0.004 | 0.268 |
| Choriocapillaris flow voids, size | 0.003 | -0.014 to 0.021 | 0.711 |

| | | | |
|---|---|---|---|
| **Superficial FAZ** | | | |
| Superficial FAZ area | 8.150 | -1.373 to 17.672 | 0.102 |
| Superficial FAZ perimeter | 2.131 | -0.098 to 4.360 | 0.069 |
| Superficial FAZ circularity | 4.103 | -6.573 to 14.778 | 0.456 |
| **Deep FAZ** | | | |
| Deep FAZ area | 2.448 | 0.131 to 4.765 | **0.045** |
| Deep FAZ perimeter | 0.908 | -0.299 to 2.115 | 0.149 |
| Deep FAZ circularity | -11.165 | -32.471 to 10.142 | 0.311 |

OCT = optical coherence tomography; RNFL = retinal nerve fibre layer; GCIPL = ganglion cell–inner plexiform layer; OCTA = OCT angiography; SCP = superficial capillary plexus; VD = vessel density; DCP = deep capillary plexus; FAZ = foveal avascular zone.

Adjusted for age and gender.

Bold values denote statistical significance at the p < 0.05 level.

**Table S2.** Correlations of OCT/OCTA parameters with overall EDSS scores at baseline.

| **Parameter** | **β** | **95% CI** | **p value** |
|---|---|---|---|
| **OCT** | | | |
| RNFL | -0.024 | -0.057 to 0.009 | 0.167 |
| GCIPL | -0.047 | -0.091 to -0.003 | **0.041** |
| **OCTA** | | | |
| **SCP VD** | | | |

| | | | |
|---|---|---|---|
| Large vessels, VD | -1.545 | -2.582 to -0.508 | **0.006** |
| Small vessels, VD | -0.177 | -0.434 to 0.079 | 0.184 |
| Total vessels, VD | -0.234 | -0.464 to -0.003 | 0.055 |
| **DCP** | | | |
| Total vessels, VD | -0.032 | -0.212 to 0.148 | 0.733 |
| **Choriocapillaris flow voids** | | | |
| Choriocapillaris flow voids, density | -0.005 | -0.267 to 0.258 | 0.972 |
| Choriocapillaris flow voids, number | -0.002 | -0.006 to 0.002 | 0.292 |
| Choriocapillaris flow voids, size | 0.005 | -0.003 to 0.013 | 0.246 |
| **Superficial FAZ** | | | |
| Superficial FAZ area | 1.622 | -2.850 to 6.094 | 0.482 |
| Superficial FAZ perimeter | 0.497 | -0.554 to 1.548 | 0.360 |
| Superficial FAZ circularity | 2.175 | -2.678 to 7.028 | 0.385 |
| **Deep FAZ** | | | |
| Deep FAZ area | -0.066 | -1.181 to 1.050 | 0.909 |
| Deep FAZ perimeter | -0.057 | -0.623 to 0.509 | 0.845 |
| Deep FAZ circularity | -2.332 | -12.153 to 7.489 | 0.644 |

OCT = optical coherence tomography; RNFL = retinal nerve fibre layer; GCIPL = ganglion cell–inner plexiform layer; OCTA = OCT angiography; SCP = superficial capillary plexus; VD = vessel density; DCP = deep capillary plexus; FAZ = foveal avascular zone.

Adjusted for age and gender.

Bold values denote statistical significance at the $p < 0.05$ level.

**Table S3.** Subgroup analysis of correlations of OCT/OCTA parameters with overall EDSS scores at baseline for patients with a visual EDSS of 0 points.

| **Parameter** | **β** | **95% CI** | **p value** |
|---|---|---|---|
| **OCT** | | | |
| RNFL | -0.056 | -0,099 to -0,013 | **0.019** |
| GCIPL | -0.099 | -0,160 to -0,038 | **0.004** |
| **OCTA** | | | |
| **SCP VD** | | | |
| Large vessels, VD | -1.574 | -2,864 to -0,284 | **0.024** |
| Small vessels, VD | 0.038 | -0,399 to 0,475 | 0.868 |
| Total vessels, VD | -0.131 | -0,541 to 0,279 | 0.537 |
| **DCP** | | | |
| Total vessels, VD | -0.267 | -608,040 to 74,040 | 0.136 |
| **Choriocapillaris flow voids** | | | |
| Choriocapillaris flow voids, density | -0.193 | -0,763 to 0,377 | 0.513 |

| | | | |
|---|---|---|---|
| Choriocapillaris flow voids, number | 0.002 | -0,004 to 0,008 | 0.638 |
| Choriocapillaris flow voids, size | -0.004 | -0,020 to 0,012 | 0.591 |
| **Superficial FAZ** | | | |
| Superficial FAZ area | 6.595 | -0,040 to 13,230 | 0.062 |
| Superficial FAZ perimeter | 1.622 | -0,075 to 3,319 | 0.072 |
| Superficial FAZ circularity | 0.398 | -7,948 to 8,744 | 0.926 |
| **Deep FAZ** | | | |
| Deep FAZ area | 0.415 | -1,594 to 2,424 | 0.688 |
| Deep FAZ perimeter | 0.123 | -0,808 to 1,054 | 0.797 |
| Deep FAZ circularity | -1.443 | -15,275 to 12,389 | 0.839 |

OCT = optical coherence tomography; RNFL = retinal nerve fibre layer; GCIPL = ganglion cell–inner plexiform layer; OCTA = OCT angiography; SCP = superficial capillary plexus; VD = vessel density; DCP = deep capillary plexus; FAZ = foveal avascular zone.

Adjusted for age and gender.

Bold values denote statistical significance at the $p < 0.05$ level.

**Table S4.** Correlations of OCT/OCTA parameters with the visual component of the EDSS scores at baseline.

| **Parameter** | **β** | **95% CI** | **p value** |
|---|---|---|---|
| **OCT** | | | |
| RNFL | 0.004 | -0.020 to 0.028 | 0.762 |

| | | | |
|---|---|---|---|
| GCIPL | -0.027 | -0.059 to 0.005 | 0.107 |
| **OCTA** | | | |
| **SCP VD** | | | |
| Large vessels, VD | -0.158 | -0.976 to 0.661 | 0.708 |
| Small vessels, VD | -0.307 | -0.467 to -0.148 | **0.001** |
| Total vessels, VD | -0.265 | -0.415 to -0.114 | **0.001** |
| **DCP** | | | |
| Total vessels, VD | -0.127 | -0.249 to -0.005 | **0.047** |
| **Choriocapillaris flow voids** | | | |
| Choriocapillaris flow voids, density | 0.072 | -0.113 to 0.258 | 0.450 |
| Choriocapillaris flow voids, number | -0.003 | -0.005 to 0.000 | 0.075 |
| Choriocapillaris flow voids, size | 0.006 | 0.001 to 0.011 | **0.032** |
| **Superficial FAZ** | | | |
| Superficial FAZ area | 2.590 | -0.510 to 5.690 | 0.110 |
| Superficial FAZ perimeter | 0.929 | 0.233 to 1.625 | **0.013** |
| Superficial FAZ circularity | 4.391 | 1.194 to 7.588 | **0.011** |
| **Deep FAZ** | | | |

| | | | |
|---|---|---|---|
| Deep FAZ area | 0.859 | 0.113 to 1.605 | **0.030** |
| Deep FAZ perimeter | 0.480 | 0.107 to 0.852 | **0.016** |
| Deep FAZ circularity | 5.873 | -0.890 to 12.636 | 0.097 |

OCT = optical coherence tomography; RNFL = retinal nerve fibre layer; GCIPL = ganglion cell–inner plexiform layer; OCTA = OCT angiography; SCP = superficial capillary plexus; VD = vessel density; DCP = deep capillary plexus; FAZ = foveal avascular zone.

Adjusted for age and gender.

Bold values denote statistical significance at the $p < 0.05$ level.

**Table S5.** Correlations between vessel density at the level of the SCP and individual functional components of the EDSS score (full cohort).

| **Parameter** | **β** | **95% CI** | **p value** |
|---|---|---|---|
| **Pyramidal** | | | |
| Large vessels, VD | -0.935 | -1.752 to -0,118 | **0.031** |
| Small vessels, VD | -0.065 | -0.263 to 0.133 | 0.525 |
| Total vessels, VD | -0.101 | -0.281 to 0.079 | 0.282 |
| **Cerebellar** | | | |
| Large vessels, VD | -0.944 | -1.775 to -0.113 | **0.032** |
| Small vessels, VD | -0.210 | -0.400 to -0.020 | **0.038** |
| Total vessels, VD | -0.239 | -0.410 to -0.068 | **0.009** |
| **Brainstem** | | | |
| Large vessels, VD | -0.089 | -0.806 to 0.628 | 0.809 |

| | | | |
|---|---|---|---|
| Small vessels, VD | -0.073 | -0.236 to 0.090 | 0.383 |
| Total vessels, VD | -0.064 | -0.215 to 0.087 | 0.411 |
| **Sensory** | | | |
| Large vessels, VD | -0.042 | -0.842 to 0.758 | 0.918 |
| Small vessels, VD | -0.124 | -0.302 to 0.054 | 0.180 |
| Total vessels, VD | -0.113 | -0.278 to 0.052 | 0.186 |
| **Bowel and bladder** | | | |
| Large vessels, VD | -0.323 | -0.878 to 0.232 | 0.262 |
| Small vessels, VD | -0.006 | -0.135 to 0.123 | 0.924 |
| Total vessels, VD | -0.034 | -0.154 to 0.086 | 0.578 |
| **Cerebral** | | | |
| Large vessels, VD | -0.282 | -0.888 to 0.324 | 0.368 |
| Small vessels, VD | -0.126 | -0.259 to 0.007 | 0.073 |
| Total vessels, VD | -0.121 | -0.244 to 0.002 | 0.062 |
| **Ambulation** | | | |
| Large vessels, VD | -1.920 | -3.216 to -0.624 | **0.006** |
| Small vessels, VD | -0.324 | -0.636 to -0.012 | **0.049** |
| Total vessels, VD | -0.383 | -0.659 to -0.107 | **0.010** |
| **Visual** | | | |

| | | | |
|---|---|---|---|
| Large vessels, VD | -0.158 | -0.976 to 0.661 | 0.708 |
| Small vessels, VD | -0.307 | -0.467 to -0.148 | **0.001** |
| Total vessels, VD | -0.265 | -0.415 to -0.114 | **0.001** |
| **Global EDSS** | | | |
| Large vessels, VD | -1.545 | -2.582 to -0.508 | **0.006** |
| Small vessels, VD | -0.177 | -0.434 to 0.079 | 0.184 |
| Total vessels, VD | -0.234 | -0.464 to -0.003 | 0.055 |

SCP = superficial capillary plexus; VD = vessel density.

Adjusted for age and gender.

Bold values denote statistical significance at the p < 0.05 level.

**Table S6.** Correlations of OCT/OCTA parameters with 9-hole peg test (9HPT) at baseline.

| **Parameter** | **β** | **95% CI** | **p value** |
|---|---|---|---|
| **9HPG dominant hand** | | | |
| **OCT** | | | |
| RNFL | 0.088 | -0.000 to 0.177 | 0.058 |
| GCIPL | -0.022 | -0.149 to 0.104 | 0.730 |
| **OCTA** | | | |
| **SCP VD** | | | |
| Large vessels, VD | -1.575 | -4.670 to 1.519 | 0.325 |
| Small vessels, VD | -0.692 | -1.373 to -0.010 | 0.054 |

| | | | |
|---|---|---|---|
| Total vessels, VD | -0.666 | -1.292 to -0.040 | **0.044** |
| **DCP** | | | |
| Total vessels, VD | -0.216 | -0.702 to 0.270 | 0.389 |
| **Choriocapillaris flow voids** | | | |
| Choriocapillaris flow voids, density | 0.413 | -0.291 to 1.117 | 0.257 |
| Choriocapillaris flow voids, number | -0.011 | -0.021 to -0.001 | **0.033** |
| Choriocapillaris flow voids, size | 0.031 | 0.012 to 0.050 | **0.003** |
| **Superficial FAZ** | | | |
| Superficial FAZ area | 2.302 | -9.951 to 14.555 | 0.715 |
| Superficial FAZ perimeter | 1.041 | -1.838 to 3.919 | 0.483 |
| Superficial FAZ circularity | 5.909 | -7.323 to 19.141 | 0.387 |
| **Deep FAZ** | | | |
| Deep FAZ area | 1.730 | -1.260 to 4.720 | 0.264 |
| Deep FAZ perimeter | 1.000 | -0.509 to 2.509 | 0.202 |
| Deep FAZ circularity | 14.108 | -12.358 to 40.574 | 0.303 |

| | | | |
|---|---|---|---|
| **9HPG non-dominant hand** | | | |

| | | | |
|---|---|---|---|
| **OCT** | | | |
| RNFL | 0.108 | -0.015 to 0.231 | 0.095 |
| GCIPL | -0.040 | -0.213 to 0.134 | 0.657 |
| **OCTA** | | | |
| **SCP VD** | | | |
| Large vessels, VD | -2.293 | -6.540 to 1.954 | 0.297 |
| Small vessels, VD | -1.568 | -2.414 to -0.722 | **0.001** |
| Total vessels, VD | -1.491 | -2.264 to -0.718 | **0.001** |
| **DCP** | | | |
| Total vessels, VD | -0.069 | -0.605 to 0.743 | 0.842 |
| **Choriocapillaris flow voids** | | | |
| Choriocapillaris flow voids, density | 0.572 | -0.396 to 1.539 | 0.254 |
| Choriocapillaris flow voids, number | -0.018 | -0.032 to -0.004 | **0.013** |
| Choriocapillaris flow voids, size | 0.045 | 0.019 to 0.071 | **0.002** |
| **Superficial FAZ** | | | |
| Superficial FAZ area | -5.150 | -21.945 to 11.645 | 0.552 |
| Superficial FAZ perimeter | 0.688 | -3.291 to 4.666 | 0.737 |

| | | | |
|---|---|---|---|
| Superficial FAZ circularity | 18.349 | 0.947 to 35.751 | **0.046** |
| **Deep FAZ** | | | |
| Deep FAZ area | 1.347 | -2.812 to 5.506 | 0.530 |
| Deep FAZ perimeter | 0.854 | -1.250 to 2.957 | 0.431 |
| Deep FAZ circularity | 15.250 | -21.342 to 51.841 | 0.419 |

OCT = optical coherence tomography; RNFL = retinal nerve fibre layer; GCIPL = ganglion cell–inner plexiform layer; OCTA = OCT angiography; SCP = superficial capillary plexus; VD = vessel density; DCP = deep capillary plexus; FAZ = foveal avascular zone.

Adjusted for age and gender.

Bold values denote statistical significance at the p < 0.05 level.

**Table S7.** Correlations of OCT/OCTA parameters with MoCA scores at baseline.

| **Parameter** | **β** | **95% CI** | **p value** |
|---|---|---|---|
| **OCT** | | | |
| RNFL | 0.015 | -0.104 to 0.134 | 0.807 |
| GCIPL | 0.000 | -0.162 to 0.161 | 0.996 |
| **OCTA** | | | |
| **SCP VD** | | | |
| Large vessels, VD | 1.943 | -2.017 to 5.903 | 0.342 |
| Small vessels, VD | -0.462 | -1.366 to 0.442 | 0.323 |
| Total vessels, VD | -0.320 | -1.160 to 0.520 | 0.460 |

| | | | |
|---|---|---|---|
| **DCP** | | | |
| Total vessels, VD | 0.037 | -0.590 to 0.664 | 0.908 |
| **Choriocapillaris flow voids** | | | |
| Choriocapillaris flow voids, density | -0.399 | -1.306 to 0.508 | 0.394 |
| Choriocapillaris flow voids, number | 0.010 | -0.003 to 0.024 | 0.132 |
| Choriocapillaris flow voids, size | -0.030 | -0.056 to -0.004 | **0.032** |
| **Superficial FAZ** | | | |
| Superficial FAZ area | 0.540 | -15.154 to 16.234 | 0.947 |
| Superficial FAZ perimeter | 0.205 | -3.500 to 3.910 | 0.914 |
| Superficial FAZ circularity | -2.448 | -19.522 to 14.626 | 0.780 |
| **Deep FAZ** | | | |
| Deep FAZ area | 0.607 | -3.277 to 4.491 | 0.761 |
| Deep FAZ perimeter | 0.243 | -1.729 to 2.214 | 0.811 |
| Deep FAZ circularity | 2.464 | -31.862 to 36.789 | 0.889 |

OCT = optical coherence tomography; RNFL = retinal nerve fibre layer; GCIPL = ganglion cell–inner plexiform layer; OCTA = OCT angiography; SCP = superficial capillary plexus; VD = vessel density; DCP = deep capillary plexus; FAZ = foveal avascular zone.

Adjusted for age and gender.

Bold values denote statistical significance at the $p < 0.05$ level.

**Table S8.** Correlations of OCT/OCTA parameters with SDMT scores at baseline.

| Parameter | β | 95% CI | p value |
|---|---|---|---|
| **OCT** | | | |
| RNFL | 0.370 | 0.005 to 0.735 | 0.054 |
| GCIPL | 0.242 | -0.274 to 0.759 | 0.363 |
| **OCTA** | | | |
| **SCP VD** | | | |
| Large vessels, VD | -0.105 | -13.049 to 12.840 | 0.987 |
| Small vessels, VD | 0.580 | -2.373 to 3.532 | 0.703 |
| Total vessels, VD | 0.475 | -2.253 to 3.203 | 0.735 |
| **DCP** | | | |
| Total vessels, VD | -0.436 | -2.457 to 1.585 | 0.675 |
| **Choriocapillaris flow voids** | | | |
| Choriocapillaris flow voids, density | -0.830 | -3.775 to 2.114 | 0.584 |
| Choriocapillaris flow voids, number | 0.046 | 0.004 to 0.088 | **0.037** |
| Choriocapillaris flow voids, size | -0.082 | -0.168 to 0.004 | 0.070 |
| **Superficial FAZ** | | | |

| | | | |
|---|---|---|---|
| Superficial FAZ area | -4.568 | -55.223 to 46.088 | 0.861 |
| Superficial FAZ perimeter | -1.788 | -13.739 to 10.163 | 0.771 |
| Superficial FAZ circularity | -14.644 | -69.631 to 40.342 | 0.605 |
| **Deep FAZ** | | | |
| Deep FAZ area | 0.314 | -12.243 to 12.870 | 0.961 |
| Deep FAZ perimeter | 0.284 | -6.085 to 6.654 | 0.931 |
| Deep FAZ circularity | 1.968 | -108.891 to 112.827 | 0.972 |

OCT = optical coherence tomography; RNFL = retinal nerve fibre layer; GCIPL = ganglion cell–inner plexiform layer; OCTA = OCT angiography; SCP = superficial capillary plexus; VD = vessel density; DCP = deep capillary plexus; FAZ = foveal avascular zone.

Adjusted for age and gender.

Bold values denote statistical significance at the $p < 0.05$ level.

**Table S9.** Correlations of OCT/OCTA parameters with RoAD scores at baseline.

| **Parameter** | **β** | **95% CI** | **p value** |
|---|---|---|---|
| **OCT** | | | |
| RNFL | -0.016 | -0.042 to 0.011 | 0.254 |
| GCIPL | -0.040 | -0.075 to -0.005 | **0.029** |
| **OCTA** | | | |
| **SCP VD** | | | |
| Large vessels, VD | -0.853 | -1.725 to 0.019 | 0.063 |

| | | | |
|---|---|---|---|
| Small vessels, VD | -0.151 | -0.354 to 0.052 | 0.154 |
| Total vessels, VD | -0.177 | -0.361 to 0.007 | 0.068 |
| **DCP** | | | |
| Total vessels, VD | -0.081 | -0.222 to 0.060 | 0.268 |
| **Choriocapillaris flow voids** | | | |
| Choriocapillaris flow voids, density | -0.129 | -0.334 to 0.075 | 0.223 |
| Choriocapillaris flow voids, number | -0.002 | -0.005 to 0.001 | 0.251 |
| Choriocapillaris flow voids, size | 0.000 | -0.007 to 0.006 | 0.962 |
| **Superficial FAZ** | | | |
| Superficial FAZ area | 1.604 | -1.937 to 5.145 | 0.380 |
| Superficial FAZ perimeter | 0.590 | -0.233 to 1.413 | 0.168 |
| Superficial FAZ circularity | 3.482 | -0.250 to 7.214 | 0.075 |
| **Deep FAZ** | | | |
| Deep FAZ area | 0.215 | -0.669 to 1.099 | 0.636 |
| Deep FAZ perimeter | 0.051 | -0.398 to 0.501 | 0.824 |
| Deep FAZ circularity | -1.426 | -9.240 to 6.388 | 0.723 |

OCT = optical coherence tomography; RNFL = retinal nerve fibre layer; GCIPL = ganglion cell–inner plexiform layer; OCTA = OCT angiography; SCP = superficial capillary plexus; VD = vessel density; DCP = deep capillary plexus; FAZ = foveal avascular zone.

Adjusted for age and gender.

Bold values denote statistical significance at the $p < 0.05$ level.
