# Supplementary material for: Correlations Between OCTA Parameters and Clinical Changes in Patients Newly Diagnosed with Multiple Sclerosis
Source: Diagnostics (Basel). 2026 Mar 11;16(6):828. doi: 10.3390/diagnostics16060828 (PMC13025533; doi:10.3390/diagnostics16060828)
Supplement: Supplementary file 1 [file diagnostics-16-00828-s001.zip › Supplementary Material File S3.pdf]

Supplementary File S3

Annex S1 - ROAD scoring at baseline

Variable	Category	Points
Sex	Female	0
	Male	1
Age	<30 years	0
	30 - 40 years	0
	> 40 years	1
Years since symptom onset	<2 years	0
	2 - 5 years	0
	>40 years	1
EDSS score	<1.5	0
	1.5 - 2.0	2
	≥2.5	3

Annex S2 - BREMSO scoring

Each of the risk factors for aggressive forms of RRMS is multiplied by its associated risk coefficient, and then summed to result in the final BREMSO score, as follows:

- age at time of debut (decades) (0.05);
- female sex (-1.07);
- functional systems involved at disease onset:
    - bowel/bladder dysfunction (0.93);
    - pyramidal (0.62);
    - pyramidal + sensory (0.81);
- poor recovery after symptom debut (0.52);
- number of functional systems involved at disease onset (0.32).
